# Exploring Mediation Roles of Child Screen-Viewing between Parental Factors and Child Overweight in Taiwan

**DOI:** 10.3390/ijerph17061878

**Published:** 2020-03-13

**Authors:** Yi-Ching Lin, Meng-Che Tsai, Carol Strong, Yi-Ping Hsieh, Chung-Ying Lin, Clara S. C. Lee

**Affiliations:** 1 Department of Early Childhood and Family Education, College of Education, National Taipei University of Education, Taipei 10671, Taiwan; ylin11@mail.ntue.edu.tw; 2 Department of Pediatrics, National Cheng Kung University Hospital, College of Medicine, National Cheng Kung University, Tainan 70101, Taiwan; ache93@yahoo.com.tw; 3 Department of Public Health, National Cheng Kung University Hospital, College of Medicine, National Cheng Kung University, Tainan 70101, Taiwan; carol.chiajung@gmail.com; 4 Department of Social Work, College of Nursing and Professional Disciplines, University of North Dakota, Grand Forks, ND 58202, USA; yiping66@gmail.com; 5 Department of Rehabilitation Sciences, The Hong Kong Polytechnic University, Hung Hom, Hong Kong; clara.sc.lee@polyu.edu.hk

**Keywords:** child, obesity, parent behavior, parental rule, screen-viewing

## Abstract

Children’s screen-viewing behavior is influenced by parents’ own screen-viewing hours and the parental rules set for screen-viewing time. However, whether childhood obesity is associated with these three factors has not been widely investigated in Chinese populations. We examined the relationships between parental rules, parental screen-viewing, child screen-viewing and child overweight. Questionnaires were distributed to 1300 parents who had children studying in two elementary schools in an eastern Taiwanese City (Yi-Lan). We collected the data (the final response rate was 77.7%) on children’s health states, the length of screen-viewing time, and whether parental rules of screen-viewing time have been set (*n* = 1005). Models using structural equation modeling, with controlling of age, gender, and physical activity of the participants, were carried out, to examine the mediated effect of child screen-viewing. The results of model testing showed that child screen-viewing could be a mediator in the associations between parental rule and child overweight (parental rule: coefficient = −0.18, *p* < 0.001); and between parent screen-viewing and child overweight (parent screen-viewing: coefficient = 0.072, *p* < 0.001). These findings suggested that parental factors (rules and little screen viewing time) effectively decreased the level of children’s screen-viewing time, and the child screen-viewing time could mediate the association between parental factors and child overweight in the Chinese population.

## 1. Introduction

Nowadays, increasing screen viewing time for children has lowered the amount of physical activity and increased sedentary behaviors for children. Indeed, this phenomenon has been found in studies conducted across countries (e.g., the United States, Norway, France, Australia and Israel) in children aged between 5 and 18 years old [1,2,3,4]. Based on a study observing American children aged 6 to 11 years old, screen viewing time is defined as a derived noun, describing the length of time engaged in sedentary behaviors, focusing on viewing the screens of electronic devices [2]. Meanwhile, another study indicated that, for Norwegian children aged 11 to 13-year-old, the various types of gaming, social network websites and phone apps further deepened children’s fascination for electronic products, resulting in the extended duration of children’s sedentary behaviors and reduction in intense physical activities [3]. As a result, children with more screen viewing time have less daily physical activity and this subsequently leads to physiological problems such as obesity [2].

The American Dietetic Association stated that the daily screen viewing time spent could be a potential predictor for the cause of overweight and obesity risks for children and adolescents [2]. Indeed, evidence from several countries such as the US, Australia, Italy and Ireland has indicated that children, aged between 5 and 18 years old, who are overweight and obese, shared the characteristic of having a high amount of screen viewing time [4]. Therefore, studying screen viewing time for children may provide insights to develop possible strategies to prevent childhood obesity. As electronic screen products become extremely common due to the rapid advancement of technology, parents in the United States often use TV or other electronic devices to calm their young children aged between 2 and 5 years old [5]. This may be related to parental behavior, such as their own screen viewing behavior. Children’s health behaviors develop within an ecological niche, with the family environment being a critical influence [6]. Specifically, a study conducted in the United States indicated that kindergarteners’ screen viewing behavior was strongly influenced by their living environment and parents [7]. Another Slovakian and Czech study pointed out that, for children aged between 11 and 15 years old, their parents’ habit of watching TV, the usage of screen electronic products at home, and even having a TV placed in the bedroom could prolong children’s screen viewing time [8].

On the other hand, parents sometimes set rules regarding screen viewing time for their children aged 6–13 years old (e.g., content restriction, behavior contingency, limiting television time, and restricting access) to reduce the exposure of screen viewing time [9]. Later, the control effectiveness was found in a TV reduction intervention with fourth graders, funded by CDC, which indicated that the number of children whose families set a limit for screen viewing time was 1.4 to 1.7 times more than those who were not restricted [10]. Children who have parental restriction on screen viewing time engaged in 22.8 fewer minutes of screen viewing time per day than their counterpart receiving no parental restriction [7]. Based on the data from both parents and children, parental rules were significantly associated with children’s low screen viewing time [1]. Healthy behaviors and habits are often developed during childhood. Therefore, it is helpful for children to cultivate healthy habits and stay at a healthy weight during childhood.

Although previous literature has shown that increasing parents’ attention and formulating regulations for children’s screen viewing time can be helpful to reduce the negative impact on child health, the applicability of these findings has not been tested in different cultures. The majority of the studies were conducted in the Western countries, where the family structure, life styles and parenting were very different from the East [11]. Parents from the Western countries respect children’s individuality, independence, and personal boundaries, compared to parents from the Eastern countries that tend to be more involved in their children’s lives [12]. According to a study that compared American and Chinese children from seventh to eighth grade, the phenomenon can be explained by the Chinese notion of “guan”, an adequate and accountable parenting style that involves “governing” and “caring for” children [13]. Oriental parents are expected to regulate their children’s behaviors and perceptions; thus, their children follow their parents’ advice and prioritize it over their own independence and autonomy [14]. The cultural differences are believed to have an impact on parenting behavior. Also, to our knowledge, there was very little literature with regard to the association between children and parents’ screen viewing time on child health. Therefore, assessing the associations between parental rules, parental screen viewing, and child overweight among Taiwanese participants (a sample that are used to represent the East Asian culture) can fill up the literature gap, by providing the associative evidence in the culture that has never been examined. Additionally, the prevalence of obesity is relatively high in Taiwan: a recently reported prevalence of childhood obesity in 2016 is approximately 15% [15]. Hence, understanding such associations may provide healthcare providers insights to overcome the childhood obesity problem in Taiwan. In light of the concerns, the current study aims at examining the following hypotheses: whether (1) parental rules or parental screen viewing were negatively associated with children’s overweight; (2) parental rules or parental screen viewing were positively associated with child screen viewing time; (3) any association between parental rules or parental screen-viewing and child overweight was mediated by child screen-viewing.

## 2. Materials and Methods

### 2.1. Participants and Procedures

The study was conducted in February 2016 and recruited families with convenient sampling from two elementary schools within the same neighborhood in an eastern Taiwan City (Yi-Lan). The information sheets with study purposes and consent forms were given to these students’ parents or their primary caretakers (e.g., grandparents or guardians), who understood their children’s daily routine and health condition well. For those who agreed to participate in the study, they have all signed a consent form authorizing their own participation and their children’s participation and responded to the given questionnaires concerning the children’s health states, the length of screen viewing time (on the weekday and weekend for both of the child and parent/gradient), and whether they had parental rules of screen viewing time.

**Figure 1:**
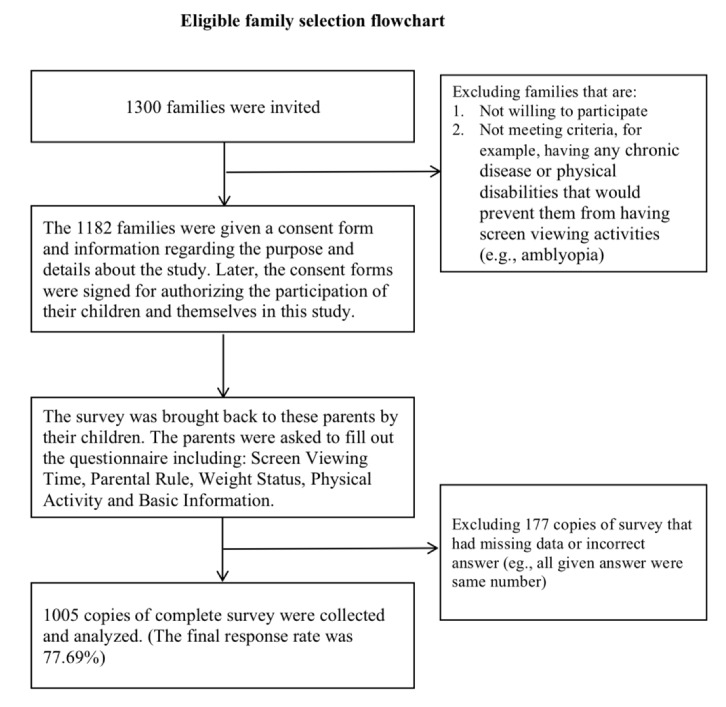
Eligible family selection Flowchart.

For eligible families, neither children nor their parents have any chronic disease or physical disabilities that would prevent them from having screen viewing activities (e.g., amblyopia). The family health information was provided by parents’ responses and the class teachers. The final response rate was 77.7% (1300 distributed and 1005 collected). The flowchart of selecting eligible families is presented below as Figure 1. The present study protocol has been approved by the Institutional Review Board (IRB) in the Research Ethics Committee of National Taiwan University Hospital (IRB Approval Case No.: 201411081RIND).

### 2.2. Measures

Screen viewing time. For accessing the level of screen viewing time, a four-question questionnaire was used to assess the usage of computer/smart phones/tablet and televisions during weekdays and weekends. The questionnaire was originally used to evaluate the screen viewing patterns of a group of 11–13-year-old Norwegian children, and had acceptable test-retest reliability (r = 0.66–0.73) [3]. Then, the internal consistency reliability of this questionnaire had been examined in Taiwanese children/parents and found to be satisfactory for all subscales and total scores (Cronbach’s α = 0.76–0.80) [14].

In the current study, the four questions were given to the parents in order to obtain the screen viewing patterns of them and their children. The questions were: (1) “How much time does your child typically spend on using the internet, reading emails, online chatting or gaming with a computer, smart phone or tablet during his/her free time on weekdays?”; (2) “How much time does your child typically spend on using the internet, reading emails, online chatting or gaming with a computer, smart phone or tablet during his/her free time on weekends?”; (3) “How much time does your child typically spend on watching TV during his/her free time in weekdays?”; (4) “How much time does your child typically spend on watching TV shows during his/her free time in weekends?” The responding options included: “<30 min”, “30 min–1 h”, “1–2 h”, “2–3 h”, “3–4 h”, “>4 h”. The internal consistency reliabilities were satisfactory for both (Cronbach’s α = 0.73 for children and 0.68 for parents).

In order to calculate the average screen viewing time of child or parent (i.e., number of hours per day), the following steps were taken. First, the duration of time spent on screen viewing each day (the choices of the questionnaire) was redefined as <30 min = 30 min; 30 min–1 h = 45 min; 1–2 h = 1.5 h; 2–3 h = 2.5 h, and 3–4 h = 3.5 h. Second, the average screen viewing time of weekdays was calculated and multiplied by 5 and that of the weekend was multiplied by 2. Finally, the sum of the average screen viewing time of weekdays and weekends was divided by 7, in order to obtain the average screen viewing time per day for child or parent. We defined “excessive screen viewing time” as >2 h per day according, to a previous study [16].

Parental Rule. Parental rule is a critical indicator of children’s screen viewing behavior, referring to the given restrictions that parents set for children when they view the screen, such as time duration. In the current study, participating parents were asked to respond to the question “Do you set a time limit for your child’s screen viewing?” If the parents’ response was “yes”, they had to answer a follow-up question by answering how long the limited screen viewing time was.

Weight status. Children’s weight and height were reported by parents who were informed semiannually by schools with children’s physical examination reports. We applied standardized measurement procedures to measure children’s height and weight. Height and weight were measured using the SECA model 207 (Seca, Hamburg, Germany) continuous display electronic scales. Body weight in kg and height in cm were measured to the nearest 0.1 unit. Children were requested to remove their shoes and extra clothes while being measured.

Children’s BMIs (calculated as weight in kilograms divided by height in meters squared) were classified into non-overweight or overweight according to specific age standard [17]. The childhood obesity expert panel from the Department of Health (DOH) has defined overweight (≥the 85th percentile value of BMI) and obesity (≥the 95th percentile value of BMI), using the gender- and age-specific criteria. The standards for age group from 6 to 13 years, with its own cut-off point for overweight and obesity, were used for this studied group. Children categorized in overweight and obesity groups were both counted as overweight.

Physical activity. The level of sufficient physical activity for children is engaging in physical activity for at least 150 min in school days, from Monday to Friday [16]. The question used to assess child physical activity was, “In the past week, how many days did your child reach 30 min (or more) of physical activities?” The options of answers ranged from 0 to 7 days. Children were categorized as having sufficient physical activity if they reported that they engaged in at least 30 min physical activity every day.

Living environment. To illustrate the content of the living environment and use to explain children’s screen-viewing behavior—the following questions were asked: “Whether or not the child own a bedroom to his/her self,” “Whether or not the child has a TV in his/her room,” “Number of computers and TVs at home.”

### 2.3. Data Analysis

**Figure 2:**
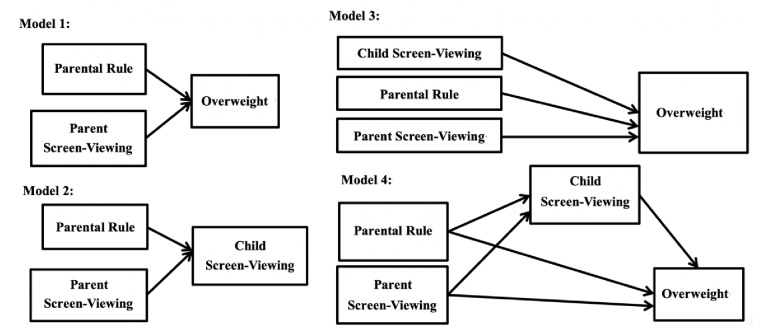
Four models test the mediated effects of child screen-viewing in the association of parental factors (parental rule and parent screen-viewing) and child overweight.

A series of models (Figure 2) were carried out to examine the mediated effect of child screen-viewing; Model 1 tested the associations between parental rule and child overweight; and between parent screen-viewing and child overweight. Model 2 tested the relationships between parental rule and child screen-viewing, and between parent screen-viewing and child screen-viewing. Model 3 tested the association between child screen-viewing and child overweight; Model 4 was an overall model that combined Models 1 to 3.

All the models were adjusted for the age, gender, and physical activity of the participants. Models 1 to 3 corresponded to the four steps on testing mediation model [18]: Model 1 tested whether there is an association that may be mediated; Model 2 tested whether the mediator is associated with the independent variables; Model 3 tested whether the mediator is correlated with the dependent variable. If Models 1 to 3 are supported, then the initial mediated effect can be determined. In addition, we applied Model 4 to additionally examine the mediated effect, through a structural equation modeling accompanied by Sobel test [19].

Except for the mediation models, which were conducted using R software under the Lavaan package, analyses were performed using SPSS 23.0 (IBM, Armonk, NY, USA) [20].

## 3. Results

The demographic data are presented on Table 1, and no significant differences were found between the two groups in their grades, family income, having a bedroom to his/her self, having a TV in the bedroom and the numbers of computer and TV at home.

In the series of models that tested for the mediated effect of child screen-viewing, Model 1 showed that both parental rule (coefficient = −0.32, *p* < 0.01) and parent screen-viewing (coefficient = 0.11, *p* < 0.001) were significantly correlated to child overweight. The associations were stronger when both factors were correlating to child screen-viewing in Model 2 (parental rule: coefficient = −1.04, *p* < 0.001; parent screen-viewing: coefficient = 0.43, *p* < 0.001). Additionally, Model 3 indicated that child screen-viewing was associated with child overweight (coefficient = 0.21, *p* < 0.001). Based on the results of Models 1 to 3, we tentatively concluded that child screen-viewing could be a mediator in the associations between parental rule and child overweight, and between parent screen-viewing and child overweight. The aforementioned mediated effect of child screen-viewing was further confirmed by our Model 4, which showed significant mediated effects (parental rule: coefficient = −0.18, *p* < 0.001; parent screen-viewing: coefficient = 0.07, *p* < 0.001) (Table 2).

## 4. Discussion

In order to examine our hypotheses, we constructed a mediation model. The mediation model indicated that child screen-viewing linked childhood obesity to parental rules and parents’ screen-viewing behaviors. The association emphasized the importance of parental factors as they impact on children’s behavior and, subsequently, children’s health. Given that child screen viewing was significantly related to child overweight [21], parental rules and appropriate screen viewing behavior may decrease the level of child screen viewing and indirectly reduce the risk of overweight for children. Moreover, significant differences were found between non-overweight and overweight groups, not by grades, but by gender.

The finding based on the difference between non-overweight and overweight groups by gender was significant. The result was in line with the previous study [22], stating that the difference could be caused by biological factors (sex differences) and society/culture (gender differences). Moreover, these aspects can extend to further behavioral patterns related to overweight such as calorie intake, physical activities and sedentary behaviors.

Our findings indicate that children with overweight, as compared with those with normal weight, received less parental rules on screen viewing, had parents who themselves had long screen-viewing hours, or were more likely to exceed two hours of screen-viewing hours per day. Our findings thus indicated that parental screen-viewing practices could have impacts on children’s body weight. We further found that parental factors and children’s screen viewing behavior were significantly associated. The association between parental factors and children’s screen viewing behavior found in our study was correspondent to preceding literature: parents’ rules toward screen viewing and their actual screen viewing behavior contribute to children’s media use [23].

Prior research indicates that children’s leisure activities are mainly arranged or decided by their parents, particularly the time and the type of activities that they are doing together [8]. Thus, we postulated that parents’ actual screen viewing behavior is considered as having the effects of role modeling to their children. Previous literature also found that parental role modeling has consistently been associated with increased similar behavior in their children [7]. Indeed, another study explains that both parents’ role modeling through low screen use and rule setting for children’s screen use can effectively deter children from participating in excessive screen viewing [24]. As a result, some researchers suggested that parents can intervene in children’s screen-viewing behaviors, either by setting rules or serving as positive role models [25].

This study contributes to the literature by bringing these associations (parents’ and children’s screen viewing time, parental rule, and childhood obesity) and examining them simultaneously, using a sample of Taiwanese children. Our findings fill the research gap and present the current trend of the impact of parental factors on children’s screen viewing behavior and health. From the practical perspective, the publicly voiced accusation that more and more parents use screens to babysit children [5] signifies that parents may either know little about the consequences of exceeding screen viewing time for child health or fail to impose screen viewing time restrictions for children [24]. The study suggested that parents should start from themselves by limiting their screen viewing time, along with practicing rules to children to develop healthy media use behavior and health. The heavier parental influence is imposed in early childhood, the less involvement would be needed by the time children reach adolescence [26].

The present study has the following strengths. First, we were able to recruit individuals from Taiwan, which shares a similar Confucianism culture to the other Great China areas (i.e., Hong Kong, Macao, and mainland China). Under Confucianism, parent-child interaction is strong and children are requested to obey their parents’ rules; this also explains our significant findings in parental rule and parent screen-viewing behavior. Therefore, our results may be generalized to other Chinese populations. Second, the mediated findings were tested using robust statistics; that is, the use of structural equation modeling with Sobel tests. Several models were proposed for examining the mediated effects; thus, the mediated effects were supported. Third, the sample size is relatively large and can thus eliminate the potential bias from outliers.

The study has some limitations. First, our analysis was cross-sectional, so we were unable to infer causality. Second, the data were collected through a self-reported questionnaire. Social desirability may lead parents to underreport the levels of screen viewing behavior for themselves and their children. Further study may include objective assessments such as accelerometers to strengthen the validity of self-reported data. Third, all the models were adjusted for age (proxy by grade), gender, and physical activity of the participants, because those variables were important factors of overweight [15,21,22]. However, socioeconomic status was not taken into account, because its influence on children’s overweight was not supported [10]. Also, because the study focused on the difference between children with and without overweight, children with underweight were not specifically excluded from the sample. Even the group size was relatively small; clear division would achieve better precision. Finally, some potential confounders were not included, such as calorie intake and parents’ weight status, which leaves us the possibility to further extend our research scope in the future.

## 5. Conclusions

The results presented that parental factors (parental rules and little parental screen viewing time) effectively decreased the level of children screen viewing time and the child screen viewing time could mediate the association between parental factors and child overweight. Results suggested that the informing strategies to promote parental factors through educational sections or intervention programs may be useful to limit child screen viewing behavior and further prevent the risk of childhood obesity.

## Figures and Tables

**Table 1:** Demographic data of participants.

Variables	*n* (%)			*X* ^2^	*p*
	Total	Non-Overweight	Overweight		
Child’s age ^a^	9.55 (1.72)				
Child’s grade				1.14	0.95
1st	178 (17.7)	144 (80.9)	34 (19.1)		
2nd	120 (11.9)	92 (76.7)	28 (23.3)		
3rd	176 (17.5)	141 (80.1)	35 (19.9)		
4th	180 (17.9)	140 (77.8)	40 (22.2)		
5th	177 (17.6)	139 (78.5)	38 (21.5)		
6th	174 (17.3)	136 (78.2)	38 (21.8)		
Gender				11.95	0.001
Boys	503 (50.0)	374 (47.2)	129 (60.6)		
Girls	502 (50.0)	418 (52.8)	84 (39.4)		
Parental Rule				20.88	<0.001
No	265 (26.4)	183 (23.1)	82 (38.7)		
Yes	739 (73.6)	609 (76.9)	130 (61.3)		
Parent screen-viewing				16.59	<0.001
>2 h per day	702 (69.9)	529 (66.8)	173 (81.2)		
<2 h per day	303 (30.1)	263 (33.2)	40 (18.8)		
Child screen-viewing				89.57	<0.001
>2 h per day	552 (54.9)	374 (47.2)	178 (83.6)		
<2 h per day	453 (45.1)	418 (52.8)	35 (16.4)		
Income ^†^				4.32	0.23
Low	97 (9.7)	73 (9.2)	24 (11.3)		
Mid-Low	345 (34.3)	262 (33.1)	83 (39.0)		
Mid	304 (30.2)	247 (31.2)	57 (26.8)		
High	259 (25.8)	210 (26.5)	49 (23.0)		
Own a Room				0.04	0.85
No	493 (49.2)	390 (49.3)	103 (48.6)		
Yes	510 (50.8)	401 (50.7)	109 (51.4)		
TV in the Room				8.25	0.004
No	904 (90.1)	724 (91.5)	180 (84.9)		
Yes	99 (9.9)	67 (8.5)	32 (15.1)		
Number of Computer at home				4.21	0.24
0	79 (7.9)	60 (7.6)	19 (9.0)		
1	487 (48.5)	384 (48.5)	103 (48.6)		
2	309 (30.8)	238 (30.1)	71 (33.5)		
>3	129 (12.8)	110 (13.9)	19 (9.0)		
Number of TV at home				6.80	0.08
0	17 (1.7)	16 (2.0)	1 (0.5)		
1	282 (28.1)	233 (29.4)	49 (23.1)		
2	388 (38.6)	303 (38.3)	85 (40.1)		
>3	317 (31.6)	240 (30.3)	77 (36.3)		

Note. ^a^ Reported in mean (SD) ^†^ Monthly household income: Low = under NTD 30,000; Mid–Low = TD 30,001–70,000; Mid = NTD 70,001–150,000; High = above NTD 150,001. 1 USD ≅ 30 NTD.

**Table 2:** Mediated roles of child screen-viewing.

Model Number:	Coefficient (SE)					R^2^
Dependent Variable	Independent Variable			Mediated Effect of Child Screen-Viewing		
	Parental Rule	Child Screen-Viewing	Parent Screen-Viewing	Parental Rule	Parent Screen-Viewing	
M1: Overweight	−0.32 (0.10) **	--	0.11 (0.02) ***	--	--	0.14
M2: Child screen-viewing	−1.04 (0.10) ***	--	0.43 (0.02) ***	--	--	0.38
M3: Overweight	−0.14 (0.11)	0.21 (0.03) ***	0.04 (0.03)	--	--	0.19
M4: Child screen-viewing	−1.03 (0.09) ***	--	0.42 (0.02) ***	--	--	0.33
M4: Overweight	−0.15 (0.10)	0.17 (0.03) ***	0.04 (0.03)	−0.18 (0.04) ***	0.07 (0.01) ***	0.19

** *p* < 0.01; *** *p* < 0.001. Note. All the models are adjusted for the age, gender, and physical activity of children.
